# Evaluation of the performance and reliability of a smartwatch capable of performing ECGs: A prospective observational study

**DOI:** 10.1016/j.heliyon.2025.e41682

**Published:** 2025-01-03

**Authors:** Ferah Kader, Burcu Bayramoglu, Ismail Tayfur

**Affiliations:** a Reyhanlı State Hospital, Department of Emergency Medicine, Hatay, Türkiye; b University of Health Sciences, Sancaktepe Sehit Prof. Dr. Ilhan Varank Training and Research Hospital, Department of Emergency Medicine, Istanbul, Türkiye

## 1 Introduction

With the widespread adoption of smartwatches, they have extensively been utilized for monitoring health-related parameters. Initially employed for purposes such as step counting, exercise measurement, and sleep monitoring, smartwatches are now being increasingly incorporated in the surveillance of vital signs, driven by advancements in technology.

Developments in both hardware and software have facilitated the involvement of smart devices in monitoring significant health parameters. The ability of individuals to monitor their own vital signs without the need for an additional device, simply by employing wearable technology products, has gained particular significance amidst the COVID-19 pandemic. The use of wearable technology products for monitoring vital signs has empowered individuals to easily access information about oxygen saturation, heart rate, and blood pressure at any time, facilitating the implementation of various preventive measures and prompting medical consultations if necessary.

Some studies on the management of the COVID-19 pandemic have suggested the use of smart devices in pre-hospital triage to alleviate the workload of hospitals. Studies related to the management of the COVID-19 pandemic have indicated that smart devices can play a role in triage prior to hospitalization, potentially reducing the burden on healthcare facilities [1]. One of the latest application areas of smartwatches involves rhythm analysis, with a notable emphasis on the detection of atrial fibrillation (AF). As advancements have been made, there has been a quest to explore the detectability of other rhythm disorders and even ECG changes alongside AF [2].

In this study, the success of the Apple Watch Series 7 WatchOS 8 model in rhythm analysis was evaluated by comparing its performance with rhythms obtained from an ECG device used in the emergency department and interpreted by experienced emergency physicians. The aim was not only to assess rhythm analysis but also to ascertain which rhythms were not evaluated and identify the circumstances that hindered the ability of the smartwatch to conduct rhythm analysis. Upon reviewing the relevant literature, similar studies were found to have been frequently conducted with hospitalized or home-monitored patients, whereas our study represents one of the initial investigations to involve patients presenting to the emergency department.

## 2 Material and method

This cross-sectional, descriptive clinical study was conducted prospectively at a tertiary care center between October 2022 and January 2023. Ethical approval for the study was obtained from the institutional ethics committee (approval number: E−46059653-020).

### 2.1 Study design and setting

**Fig. 1:**
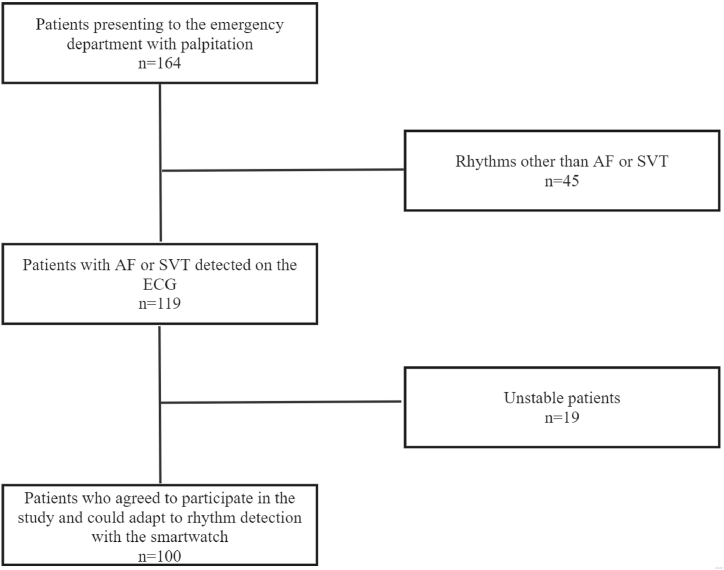
Flow chart for inclusion and exclusion criterias.

The study included 100 patients aged 18 years and older who presented to the emergency department and required an ECG evaluation, with their ECG results being interpreted by an experienced emergency physician as AF or supraventricular tachycardia (SVT). Patients under 18 years of age, those with pacemakers or implantable cardioverter-defibrillators, those requiring emergency interventions, those whose ECG rhythm was evaluated as other than AF or SVT, and those unwilling to participate were excluded from the study (Fig. 1).

**Fig. 2:**
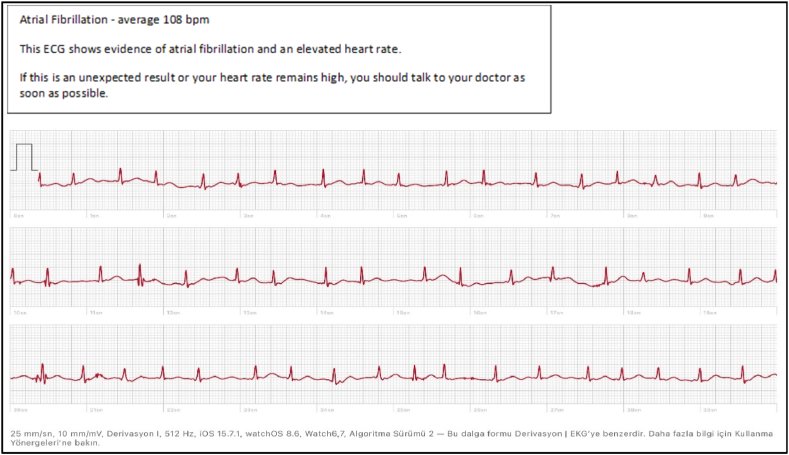
Example of the ECG recording taken with the smartwatch.

All included patients underwent a standard 12-lead ECG (25 mm/s, 10 mm/mV, 100 MHz). In addition, recordings showing at least 30 s of DI derivation were obtained at the time of admission using an FDA-approved, Apple Watch Series 7 smartwatch with the WatchOS 8 operating system [3]. Standard 12-lead ECG recordings were acquired using a GE HEALTHCARE MAC 2000 device (serial number: 2063587-001), routinely calibrated by the biomedical unit of our hospital. The ECG recordings were obtained with the smartwatch by placing the disinfected smartwatch on the left wrist of the patient in a supine position, followed by the second finger of the right hand pressing the side button on the watch for 30 s These recordings were transferred to a computer system and printed out for comparison with the reference ECG recordings, and these printouts were added to the case forms (Fig. 2).

In ECG recordings, the absence of a P wave and continuous irregularity of the rhythm were considered indicative of AF, while recordings exhibiting narrow QRS complexes (QRS duration <120 ms), regularity, and heart rates exceeding 120 beats per minute were classified as SVT [4]. ECG recordings obtained with the reference ECG device for the diagnosis of AF were evaluated by emergency medicine specialists, and the rhythm interpretation provided by the smartwatch was taken into consideration for ECG recordings obtained with the smartwatch. Prior to the study, it was determined that according to the smartwatch interpretation, recordings of patients with the SVT rhythm were labeled as “high heart rate”, whereas those with sinus tachycardia were labeled as “sinus rhythm”.

### 2.2 Statistical analysis

Descriptive statistics (mean and standard deviation) were used to describe continuous variables. The relationship between pulse measurements obtained with the reference ECG device and the smartwatch was assessed using the dependent-samples *t*-test, correlation analysis, and the Bland-Altman method. Additionally, the sensitivity, specificity, positive predictive value (PPV), and negative predictive value (NPV) of the measurements obtained with the smartwatch in detecting rhythm disorders were calculated. Regression analysis was employed to explore the reasons for the inability of the smartwatch to detect rhythm. The statistical significance level was set at p < 0.05. In addition, a confidence interval criterion of 95 % was applied. Statistical analyses were performed using the Statistical Package for the Social Sciences (SPSS) for Windows v. 22.0 (IBM Corporation, Chicago, Illinois).

## 3 Results

The study was completed with the participation of 100 patients. The mean age of the participants was 70.1 ± 14.10 years, with females constituting 60 % of the sample. The relationship between the measurements obtained with the reference ECG device and the smartwatch was evaluated using the dependent samples *t*-test and correlation analysis. Additionally, the sensitivity, specificity, PPV, and NPV of the measurements obtained with the smartwatch in detecting rhythm disorders were calculated.

**Fig. 3:**
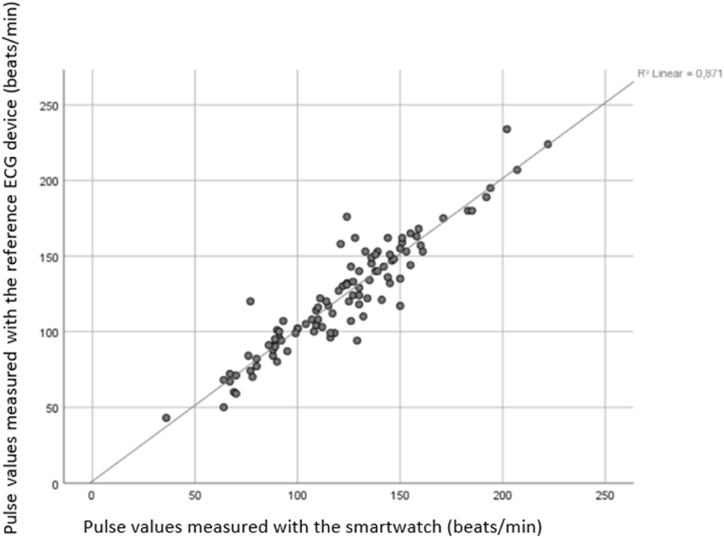
Correlation between the heart rate measurements performed by the reference ECG device and the smartwatch.

Pulse measurements obtained by the reference ECG device and the smartwatch were evaluated using the dependent-samples *t*-test. Accordingly, the mean pulse measurement obtained by the reference ECG device was found to be 123.27 ± 37.304 beats/min, while the mean pulse measurement obtained by the smartwatch was 121.87 ± 34.773 beats/min (Table 1). No significant difference was observed between the measurements obtained by the smartwatch and the reference ECG device (p = 0.299). The relationship between the pulse measurements obtained by the reference ECG device and those obtained by the smartwatch was also examined through a correlation analysis, which revealed a significant positive correlation (p < 0.001, r = 0.933) (Fig. 3).

**Table 1:** Comparison of the measurements performed with the ECG device and the smartwatch.

Measurement device	Mean	SD	t	Sig. (2-tailed)
ECG device (beat/min)	123.27	37.304	1.045	0.299
Smartwatch (beat/min)	121.87	34.773

**Fig. 4:**
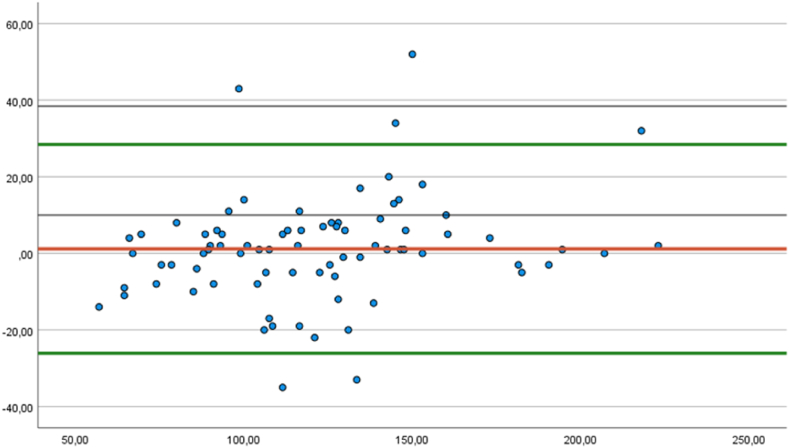
Bland-Altman plot of the agreement between the pulse values measured by the reference ECG device and the smartwatch.

The agreement between the pulse values measured by the reference ECG device and the smartwatch was evaluated using the Bland-Altman method. The mean difference for the measurements was 1.40 (95 % confidence interval: −1.2594-4.0594), with a standard deviation of 13.4. The limits of agreement at the 95 % confidence level were calculated using the mean and standard deviation values of the differences. The upper limit for deviations in the measurements was found to be 27.664, and the lower limit was −24.864 (Fig. 4).

When evaluating the heart rhythms assessed by the reference ECG device, AF was detected in 86 patients (86 %), and SVT in 14 (14 %). Upon evaluating the heart rhythms recorded by the smartwatch, AF was detected in 65 patients (65 %), and a high heart rate in 14 (14 %). It was observed that all patients identified as having SVT on the ECG device were also interpreted as having a high heart rate by the smartwatch. Additionally, one patient (1 %) whose rhythm was assessed as AF by the clinician based on the reference ECG recording was reported to have a sinus rhythm by the smartwatch. The rhythm of 20 patients (20 %) could not be evaluated by the smartwatch. Among these patients, seven (35 %) had poor recordings, eight (40 %) had high heart rates, and one (5 %) had a low heart rate, and the device was not able to detect rhythm in the remaining four (20 %) (Table 2).

**Table 2:** Pulse and rhythm analysis.

Arrhythmia	ECG measurement		Smartwatch measurement	
	n	%	n	%
AF	86	86,0	65	65,0
SVT/high heart rate	14	14,0	14	14,0
Sinus rhythm	–	–	1	1,0
Inconclusive	–	–	20	20,0
Total	100	100	100	100

AF: atrial fibrillation SVT: supraventricular tachycardia.

Predictive factors for the inability to perform rhythm analysis with the smartwatch were evaluated using a binary logistic regression analysis. This evaluation revealed that in patients with tachycardia (>100 beats/min), the likelihood of performing rhythm analysis with the smartwatch was 0.202 times lower compared to those with normal heart rates (odds ratio [OR] = 0.202, p = 0.026). Additionally, the use of antiarrhythmic medication was identified as a factor that reduced the likelihood of performing heart rhythm analysis with the smartwatch (OR = 0.243, p = 0.034). The analysis concluded that variables such as hypertension, a history of coronary artery disease, and age were not significant predictors of the ability of the smartwatch to perform rhythm analysis (p > 0.05) (Table 3).

**Table 3:** Predictive factors for the smartwatch evaluation of heart rhythm analysis.

Variables	B	S.E.	Wald	OR	Sig.	95.0 % CI	
						Lower	Upper
Pulse							
>100 beats/min	−1.600	0.718	4.969	0.202	0.026	0.049	0.824
≤100 beats/min						Reference	
Antiarrhythmic medication use							
Present	−1.415	0.669	4.481	0.243	0.034	0.065	0.900
Absent						Reference	
Hypertension							
Present	−2.137	1.089	3.852	0.118	0.050	0.014	0.997
Absent						Reference	
CAD							
Present	0.415	0.588	0.499	1.514	0.480	0.479	4.789
Absent						Reference	
Age (years)							
>70	−0.127	0.549	0.054	0.880	0.817	0.300	2.582
≤70						Reference	
Constant	5.305	1.371	14.975	201.414	0.000		

CAD:coronary artery disease, OR=Odds Ratio, CI=Confidence Interval, Cox&Snell R^2^=0.144, Nagelkerke R^2^=0.227, Accuracy=0.80, χ2=84.547, p=0.008.

## 4 Discussion

In our study, evaluation of the success of the smartwatch in detecting AF, it had a sensitivity of 75.5 %, a specificity of 100 %, a PPV of 100 %, and an NPV of 40 %. For rhythms identified as SVT and labeled as a high heart rate by the smartwatch, the sensitivity, specificity, PPV, NPV were all found to be 100 %. Because of these results, we can conclude that this smartwatch yielded similar results to the reference ECG device in pulse measurements and demonstrated success in rhythm detection.

In 2022, it was estimated that the global population of smartwatch users surpassed 216 million, with projections indicating an increase to over 230 million by 2026 [5]. Manufacturers of smartwatch technology commenced the development of health-oriented products for users in the early 2010s [6]. However, the extent to which health applications on smartwatches are genuinely beneficial remains a topic of ongoing debate. The premise of this study was to investigate the performance of these devices in analyzing heart rhythms and their potential for everyday use.

A study by Theurl et al. conducted with 263 patients using the Garmin vivoactive 4, a device that employs photoplethysmography (PPG) technology to measure heart rate, revealed a high level of agreement between the heart rate calculated by the smartwatch and that derived from the ECG device [7]. Similarly, in our study, no significant difference was observed between the pulse rates calculated by the smartwatch and the reference ECG device, with a significant positive correlation noted between the two values.

Selder et al. conducted a study on 151 patients using the Biostrap smart wristband and Fitbit smartwatch. The authors compared the ECG data obtained from 78 patients with 156 measurements from Biostrap and 73 patients with 143 measurements from Fitbit. The sensitivity, specificity, PPV, NPV, and accuracy were found to be 98 % vs. 97 %, 96 % vs. 100 %, 96 % vs. 100 %, 99 % vs. 97 %, and 97 % vs. 99 % for Biostrap and Fitbit, respectively [8]. In another study undertaken by Mannhart et al. with 117 patients, various smartwatches, including Apple Watch 6, AliveCor Kardia Mobile or Kardia Mobile 6L, Fitbit Sense, Samsung Galaxy Watch 3, and Withings ScanWatch, were used, and the data obtained from smartwatches were compared with automated interpretations of 12-lead ECGs, interpretations by physicians, and interpretations by artificial intelligence. The sensitivity and specificity values of the smartwatch software for AF detection were respectively found to be 100 and 97 % for Apple, 96 and 100 % for Fitbit, 100 and 98 % for AliveCor, 100 and 93 % for Samsung, and 96 and 95 % for Withings. Consequently, all five devices were found to have high accuracy in diagnosing AF [9]. Wasserlauf et al., using the Apple Watch Series 5 in a study with 30 participants, reported receiving irregular rhythm notifications in eight out of 11 cases with a known diagnosis of AF during a six-month follow-up period. On a participant basis, the smartwatch demonstrated 72 % sensitivity, 100 % specificity, 100 % PPV, and 90 % NPV in detecting rhythm disorders [10]. In a study by Mannhart et al. with 201 patients comparing the Apple Watch Series 6, Samsung Galaxy Watch 3, Withings ScanWatch, Fitbit Sense, and AliveCor Kardia Mobile, the sensitivity and specificity values were respectively found to be 85 % and 75 % for Apple Watch 6, 85 % and 75 % for Samsung Galaxy Watch 3, 58 % and 75 % for Withings ScanWatch, 66 % and 79 % for Fitbit Sense, and 79 % and 69 % for AliveCor Kardia Mobile [11]. Pepplinkhuizen et al. conducted a study on 74 patients using the Apple Watch Series 6. The sensitivity for detecting AF was found to be 93.5 %, and the specificity for detecting sinus rhythm was found to be 100 %. The PPV was 100 %, and the NPV was 94 %. When patients with pacemaker rhythms were excluded, the sensitivity for AF detection increased to 97.5 %. Thus, the study concluded that smartwatches exhibited high accuracy in detecting AF and sinus rhythm [12]. In a study by Guo et al., examining data from 187,912 individuals using Huawei Watch GT (version 1.0.3.52 or higher), Honor Watch (version 1.0.3.52 or higher), and Honor Band 4 (version 1.0.0.86 or higher), suspicious AF episodes were detected in 424 (0.2 %) cases. The ECG data from 262 patients were analyzed, with 227 ultimately receiving a confirmed diagnosis of AF. The PPV of smart devices for AF diagnosis was found to be 91.6 % [13]. In brief, these studies indicate that the sensitivity values of smartwatches or wristbands for rhythm analysis range from 58 % to 100 %, specificity values from 69 % to 100 %, PPV from 91.6 % to 100 %, and NPV from 90 % to 99 %. In our study, utilizing an Apple Watch Series 7 smartwatch, the sensitivity, specificity, PPV, and NPV for detecting AF were 75.5 %, 100 %, 100 %, and 40 %, respectively, while these values were all 100 % for detecting SVT represented by a high heart rate. It is notable that the results of our study, particularly in terms of sensitivity and specificity, align closely with existing literature.

In a study conducted by Selder et al., it was observed that rhythm analysis could not be performed for 19 (12 %) cases measured by the Biostrap smart wristband and 7 (5 %) cases measured by the Fitbit smart wristband. In cases where the rhythm could not be evaluated, statistically significant higher incidences of peripheral vascular diseases and direct oral anticoagulant use were found, while the use of verapamil was associated with a statistically significant lower incidence [8]. Mannhart et al. determined that 16 % of measurements from Apple, 15 % from Samsung, 26 % from Withings, 19 % from Fitbit, and 23 % from AliveCor were reported as inconclusive. Among the rhythms reported as inconclusive, 13 % exhibited low-quality data, 20 % showed bradycardia, and 8 % displayed tachycardia, while no specific cause could be identified for the remaining 59 % of cases [9]. In another study by Mannhart et al., 18 % of rhythms could not be analyzed by Apple Watch 6, 17 % by Samsung Galaxy Watch 3, 24 % by Withings ScanWatch, 21 % by Fitbit Sense, and 26 % by AliveCor Kardia Mobile [11]. Pepplinkhuizen et al. identified premature atrial contractions, premature ventricular contractions, low amplitude, isoelectric line shift, and noise as reasons for rhythms that could not be determined by smartwatches. Similar findings were observed in cases evaluated as poor recordings, with the cause not being able to be identified in one case [12]. In another study involving the same brand and model device as in our study, rhythm analysis could not be conducted in 86 (8 %) of 1073 patients. Of these cases, 49 were attributed to poor recordings, while 37 were due to a high or low heart rate. Among the warnings given for rhythms that cannot be evaluated by the smartwatch, the main reasons for “poor recording” are noise, artifacts, and low signal quality. When a patient's heart rate exceeds 120 beats per minute or drops below 50, the measurement capacity of the smartwatch significantly decreases, and the possibility of unevaluated rhythms increases, as indicated in the smartwatch application [14].

In the above-mentioned studies, the rate of cases where rhythm analysis could not be performed ranges from 5 % to 26 %. Although the specific rate varies among studies, common reasons include low-quality data and high or low heart rates. In our study, the rate of cases where rhythm analysis could not be performed by the smartwatch was 20 %. Of these cases, 35 % were attributed to poor recordings, 40 % to a high heart rate, and 5 % to a low heart rate, while the remaining 20 % were inconclusive. These findings from our study are consistent with the literature. Unlike the study by Selder et al., our study also identified antiarrhythmic medication as a factor reducing the ability of the smartwatch to perform rhythm analysis.

## 5 Conclusion

In our study using the Apple Watch Series 7 with WatchOS 8 for pulse and rhythm analyses, we found that this smartwatch yielded similar results to the reference ECG device in pulse measurements and demonstrated success in rhythm detection. “Poor recording” results obtained in measurements with smartwatches are considered to stem from software deficiencies and shortcomings in sensor technologies. Improvements in software algorithms could lead to more accurate evaluations of heart rhythm analyses.

With current technologies, ECG recordings taken as a single derivation (DI) can only facilitate the diagnosis of AF and other rhythms. In the future, the development of electrode/sensor technologies that can be placed on extremities or even on the anterior chest wall and connected to smart devices may enable the acquisition of 12-lead ECG recordings with smartwatches.

Considering the rapid proliferation and advancement of wearable technologies, it is believed that they will become indispensable technologies for both healthcare settings and daily life. As smart devices become more integrated with health information systems, patients' health data can be shared instantly with healthcare institutions. Particularly in emergencies, the integration of patients' vital signs through integrated health information systems can greatly contribute to pre-hospital triage assessments. Similarly, determining the cardiac urgency of patients through rhythm analysis can enable ambulances and healthcare facilities to start preparations for patients much earlier. It is expected that artificial intelligence will also soon be involved in the rapid development and transformation of healthcare technologies. With the integration of artificial intelligence, a patient's pre-hospital health status can be assessed, and artificial intelligence can simultaneously provide recommendations to the patient and share the patient's health status, location, and urgency with the most appropriate healthcare institution. These technological advancements indicate the beginning of a major transformation in healthcare technology. Studies in this field can accelerate the integration of wearable devices into healthcare systems and contribute to this transformation.

## Limitations

The patient population was determined to be 100 individuals. Differences in rhythm analyses could be better observed with a larger number of patients. In addition, the ability of the smartwatch to only obtain ECG recordings with DI derivation poses a limitation for rhythm analysis. Compliance issues, such as patients’ remaining still and the study conducted under emergency service conditions, during smartwatch use are other limitations of the study. Lastly, the study was conducted at a single center, which can be considered a limitation.

## CRediT authorship contribution statement

**Ferah Kader:** Writing – original draft, Methodology, Investigation, Formal analysis, Data curation. **Burcu Bayramoglu:** Writing – review & editing, Writing – original draft, Resources, Methodology, Investigation, Formal analysis. **Ismail Tayfur:** Writing – review & editing, Writing – original draft, Supervision, Resources, Project administration, Methodology, Investigation, Formal analysis, Conceptualization.

## Funding

None.

## Declaration of competing interest

The authors declare that they have no known competing financial interests or personal relationships that could have appeared to influence the work reported in this paper.

## References

[1] Xie W., Cao X., Dong H., Liu Y. (2019 Nov 11). The use of smartphone-based triage to reduce the rate of outpatient error registration: cross-sectional study. JMIR MHealth UHealth.

[2] Van Der Zande J., Strik M., Dubois R., Ploux S., Alrub S.A., Caillol T. (2023 Feb 25). Using a smartwatch to record precordial electrocardiograms: a validation study. Sensors.

[3] Food and Drug Administration (2018).

[4] Lundqvist C.B., Potpara T.S., Malmborg H. (2017 Jun). Supraventricular arrhythmias in patients with adult congenital heart disease. Arrhythmia Electrophysiol. Rev..

[5] Statista [Internet]. [cited 2023 April 8]. Global: smartwatch users 2017-2026. Available from: https://www.statista.com/forecasts/1314339/worldwide-users-of-smartwatches?locale=en.

[6] Reeder B., David A. (2016 Oct 1). Health at hand: a systematic review of smart watch uses for health and wellness. J. Biomed. Inf..

[7] Theurl F., Schreinlechner M., Sappler N., Toifl M., Dolejsi T., Hofer F. (2023 Jun 1). Smartwatch-derived heart rate variability: a head-to-head comparison with the gold standard in cardiovascular disease. Eur Heart J - Digit Health.

[8] Selder J.L., Te Kolste H.J., Twisk J., Schijven M., Gielen W., Allaart C.P. (2023 May 26). Accuracy of a standalone atrial fibrillation detection algorithm added to a popular wristband and smartwatch: prospective diagnostic accuracy study. J. Med. Internet Res..

[9] Mannhart D., Lefebvre B., Gardella C., Henry C., Serban T., Knecht S. (2023 May). Clinical validation of an artificial intelligence algorithm offering cross-platform detection of atrial fibrillation using smart device electrocardiograms. Arch Cardiovasc Dis.

[10] Wasserlauf J., Vogel K., Whisler C., Benjamin E., Helm R., Steinhaus D.A. (2023 May). Accuracy of the Apple watch for detection of AF: a multicenter experience. J. Cardiovasc. Electrophysiol..

[11] Mannhart D., Lischer M., Knecht S., Lavallaz J du F de, Strebel I., Serban T. (2023). Clinical validation of 5 direct-to-consumer wearable smart devices to detect atrial fibrillation: BASEL wearable study. JACC Clin Electrophysiol..

[12] Pepplinkhuizen S., Hoeksema W.F., van der Stuijt W., van Steijn N.J., Winter M.M., Wilde A.A.M. (2022 Dec 15). Accuracy and clinical relevance of the single-lead Apple Watch electrocardiogram to identify atrial fibrillation. Cardiovasc Digit Health J.

[13] Guo Y., Wang H., Zhang H., Liu T., Liang Z., Xia Y. (2019 Nov). Mobile photoplethysmographic technology to detect atrial fibrillation. J. Am. Coll. Cardiol..

[14] Apple_Watch_Arrhythmia_Detection.pdf [Internet]. [cited 2023 May 10]. Available from: https://www.apple.com/healthcare/docs/site/Apple_Watch_Arrhythmia_Detection.pdf.

